# Nomogram for predicting pathological complete response and tumor downstaging in patients with locally advanced rectal cancer on the basis of a randomized clinical trial

**DOI:** 10.1093/gastro/goz073

**Published:** 2020-02-08

**Authors:** Jian-Wei Zhang, Yue Cai, Xiao-Yu Xie, Hua-Bin Hu, Jia-Yu Ling, Ze-Hua Wu, Ping Lan, Xiao-Jian Wu, Mei-Jin Huang, Hui Wang, Liang Kang, Zhi-Yang Zhou, Jian-Ping Wang, Yan-Hong Deng

**Affiliations:** 1 Department of Medical Oncology, The Sixth Affiliated Hospital of Sun Yat-sen University, Guangzhou, Guangdong, P. R. China; 2 Guangdong Provincial Key Laboratory of Colorectal and Pelvic Floor Diseases, Guangzhou, Guangdong, P. R. China; 3 Department of Colorectal Surgery, The Sixth Affiliated Hospital of Sun Yat-sen University, Guangzhou, Guangdong, P. R. China; 4 Department of Radiology, The Sixth Affiliated Hospital of Sun Yat-sen University, Guangzhou, Guangdong, P. R. China

**Keywords:** nomogram, pathological complete response, tumor downstaging, locally advanced rectal cancer

## Abstract

**Background:**

Preoperative fluoropyrimidine with radiotherapy was regarded as the standard of care for locally advanced rectal cancer (LARC). The model for predicting pCR in LARC patients was based on standard treatment only. This study aimed to establish a nomogram with pretherapeutic parameters and different neoadjuvant regimens for predicting pathologic complete response (pCR) and tumor downstaging or good response (ypT0-2N0M0) after receiving neoadjuvant treatment in patients with LARC based on a randomized clinical trial.

**Methods:**

Between January 2011 and February 2015, 309 patients with rectal cancer were enrolled from a prospective randomized study (NCT01211210). All pretreatment clinical parameters were collected to build a nomogram for predicting pCR and tumor downstaging. The model was subjected to bootstrap internal validation. The predictive performance of the model was assessed with concordance index (C-index) and calibration plots.

**Results:**

Of the 309 patients, 53 (17.2%) achieved pCR and 132 (42.7%) patients were classified as tumor downstaging with ypT0-2N0M0. Based on the logistic-regression analysis and clinical consideration, tumor length (*P* = 0.005), tumor circumferential extent (*P* = 0.036), distance from the anal verge (*P* = 0.019), and neoadjuvant treatment regimen (*P* < 0.001) showed independent association with pCR following neoadjuvant treatment. The tumor length (*P* = 0.015), tumor circumferential extent (*P* = 0.001), distance from the anal verge (*P* = 0.032), clinical T category (*P* = 0.012), and neoadjuvant treatment regimen (*P* = 0.001) were significantly associated with good tumor downstaging (ypT0-2N0M0). Nomograms were developed to predict the probability of pCR and tumor downstaging with a C-index of 0.802 (95% confidential interval [CI], 0.736–0.867) and 0.730 (95% CI, 0.672–0.784). Internal validation revealed good performance of the calibration plots.

**Conclusions:**

The nomogram provided individual prediction responses to different preoperative treatment for patients with rectal cancer. This model might help physicians in selecting an optimized treatment, but warrants further external validation.

## Introduction

For stage II/III rectal-cancer patients, preoperative fluoropyrimidine with radiotherapy followed by total mesorectal excision (TME) surgery has been regarded as the standard treatment [1–3]. In the era of TME, preoperative chemoradiotherapy reduced only the risk of local recurrence, but no survival benefits were observed after long-term follow-up [4]. Approximately 30% of patients still developed distant metastasis [1, 5], which remains the main obstacle for improving survival of locally advanced rectal cancer (LARC). Besides, radiation exposure caused toxicities, such as anal mucous loss, sexual dysfunction, and bowel dysfunction, which have still remained the main concerns [6–8].

To improve the survival of LARC and reduce the toxicities caused by radiation exposure in patients, a phase III clinical trial comparing mFOLFOX6 with or without radiation vs fluorouracil with radiation as neoadjuvant treatment in LARC patients has been conducted [9]. This is the first study to conduct a comparative investigation of these neoadjuvant regimens and the largest to date of neoadjuvant chemotherapy alone without radiation for LARC. The initial results revealed that mFOLFOX6 with chemoradiotherapy led to pathological complete response (pCR) rate of 27.5%. Perioperative mFOLFOX6 chemotherapy alone demonstrated a lower pCR rate than chemoradiotherapy but results in a similar downstaging rate as fluorouracil-radiotherapy with less toxicity and fewer post-operative complications. Most importantly, the 3-year disease-free survival among the three arms after long-term follow-up showed no statistical significance. These promising results suggested that, in the era of TME and high-quality magnetic resonance imaging (MRI), radiotherapy might be omitted in selected patients. Different neoadjuvant treatment strategies could be considered in clinical practice.

Hence, to make the best decision for each individual patient, an accurate predictive model with baseline parameters was warranted to be established. This study aimed to predict the probability of response under different neoadjuvant treatment regimens at initial diagnosis.

## Patients and materials

### Study population

Rectal-cancer patients who underwent neoadjuvant therapy and curative resection were enrolled in this phase III randomized trial (NCT01211210). All patients were aged >18 years with histopathologically confirmed rectal adenocarcinoma and an inferior margin no more than 12 cm above the anal verge as assessed by MRI or CT.

The pretreatment clinical parameters were prospectively collected, which included age, sex, clinical TNM （8th edition）stage (MRI-based), tumor length (the distance from the inferior margin to the superior margin of the tumor), tumor circumferential extent (defined as the transverse size of the tumor as measured by an endoscope), and distance from the tumor inferior margin to the anal verge. The blood biomarkers including blood routine test (white blood cell count [WBC], hemoglobin, lymphocyte, neutrophil, and monocyte), blood biochemistry (alanine transaminase [ALT], aspartate aminotransferase [AST], total bilirubin, direct bilirubin, serum creatinine), and the serum tumor markers (carcinoembryonic antigen [CEA] and carbohydrate antigen 19–9 [CA19-9]) were also analysed. From January 2011 to February 2015, complete data of 309 patients with rectal cancer who received concurrent chemoradiotherapy or chemotherapy alone were available in this study.

### Neoadjuvant treatment regimen

The efficacy of fluorouracil chemoradiotherapy followed by TME and perioperative mFOLFOX6 with or without radiotherapy for patients with LARC was compared in this phase III study. Hence, three regimens were included in this trial.

Radiotherapy was delivered at 1.8–2.0 Gy/day with five fractions per week for a total of 23–28 fractions over 5–6 weeks and a total dose of 46.0–50.4 Gy. Surgery was performed 6–8 weeks after completing of radiation. Patients were randomly assigned to receive preoperative treatment with five cycles of de Gramont regimen with concurrent radiotherapy during cycles 2–4 and post-operative adjuvant chemotherapy with seven cycles of fluorouracil (fluorouracil-radiotherapy group). Patients in the mFOLFOX6-radiotherapy group received mFOLFOX6 for five cycles with concurrent radiotherapy during cycles 2–4 and adjuvant treatment with seven cycles of mFOLFOX6. Another group received mFOLFOX6 alone for four to six cycles and six to eight cycles of adjuvant chemotherapy.

### Pathological assessment

Two pathologists who were blinded to the clinical outcomes of the patients assessed all the resection specimens according to the American Joint Committee on Cancer (AJCC) TNM staging category (the y prefix indicated classification after neoadjuvant treatment) independently. pCR was defined as absence of any viable adenocarcinomatous cells in the resected specimens and no lymph-node metastasis (ypT0N0M0). For patients with pCR, tissue blocks were taken from the entire tumor site to confirm the absence of viable tumor cells. After neoadjuvant treatment, ypT0-2N0M0 was classified as tumor downstaging or good response.

All patients completed a written informed consent form before entering the study. The study was approved by the local medical ethics committee and was conducted in accordance to the Declaration of Helsinki and good clinical practice.

### Statistical analysis

Univariate analysis was performed using Chi-square test or Fisher’s exact test for categorical variables and student’s *t*-test for continuous variables. Significant variables with *P-*values of <0.05 entered into multivariate analyses via a logistic-regression model to identify the predictors of pCR and good response. Statistical analyses to identify independent prognostic factors were conducted using SPSS 23.0 for Windows (SPSS, Chicago, IL). Based on the results of multivariate analysis, a nomogram was formulated using R 2.13.1 (http://www.r-project.org) with survival and rms package.

The performance of nomograms for predicting the outcomes was evaluated by calculating the Harrell’s concordance index (C-index). The value of the C-index ranged from 0.5 to 1.0, in which 0.5 indicates a random chance and 1.0 indicates a perfect ability to accurately discriminate the outcome with the model. Calibration of the nomogram for pCR and good response were performed by comparing the predicted probability and the actual status after correcting the bias. Statistical significance was accepted at *P* < 0.05.

## Results

### Clinicopathologic characteristics of patients

A total of 309 patients were included in this study, with the median age of 55 years (range, 22–77 years). Table 1 presents the clinicopathologic characteristics of all patients. Approximately 67% of patients were male and 65.5% of patients had stage III rectal cancer. Of the 309 patients, 91 received de Gramont regimen with concurrent radiotherapy, 99 were assigned to the mFOLFOX6-radiotherapy group, and 119 received mFOLFOX6 chemotherapy alone as neoadjuvant treatment. All patients underwent TME surgery. The pCR rate of the whole group of patients was 17.2% (53/309) and good response with tumor downstaging to ypT0-2N0M0 was observed in 132 (44.7%) patients.

**Table 1.** Clinicopathologic characteristics of 309 patients with locally advanced rectal cancer

Parameter	No. of patients (%)
Median age, years (range)	55 (21–77)
Sex	
Male	207 (67.0)
Female	102 (33.0)
Clinical tumor category (cT)	
T2	6 (1.9)
T3	251 (81.3)
T4	52 (16.8)
Clinical nodal category (cN)	
N0	79 (25.6)
N1	125 (40.5)
N2	105 (33.9)
TNM staging	
Stage II	79 (34.5)
Stage III	230 (65.5)
CEA, ng/mL	
Median	2.73
Range	0.5–200.2
Tumor length, cm	
Median	4.0
Range	1.2–11.5
Distance from anal verge, cm	
Median	5.5
Range	1.4–12
Tumor circumferential extent, circle	
Median	0.75
Range	0.25–1
MRF involvement	
Positive	97 (31.4)
Negative	212 (68.6)
Radiotherapy	
Yes	192 (62.1)
No	117 (37.9)
Chemotherapy	
5-Fluorouracil	92 (29.8)
mFOLFOX6	217 (70.2)
pCR	
Yes	55 (17.8)
No	254 (82.2)
Tumor downstaging (ypT0-2N0)	
Yes	138 (44.7)
No	171 (55.3)

pCR, pathologic complete response; RT, radiotherapy; CEA, carcinoembryonic antigen; MRF, mesorectal fascia.

### Independent prognostic factors of early efficacy

Univariate analysis was performed on all collected variables. The results revealed that tumor length (*P* < 0.001), tumor circumferential extent (*P* = 0.005), distance from the anal verge (*P* < 0.001), and the neoadjuvant treatment regimen (*P* < 0.001) were considered significant predictors for pCR. The other factors such as CEA (*P* < 0.001), tumor length (*P* < 0.001), tumor circumferential extent (*P* < 0.001), clinical T category (*P* < 0.001), the neoadjuvant regimen (*P* = 0.007), and the distance from the anal verge (*P* = 0.031) were considered significant predictors for good response (Table 2). The shorter tumor length, smaller tumor circumferential extent, lower tumor location, lower CEA level, and radiation were associated with higher probability of response. When applied to logistic regression, tumor length (odds ratio [OR], 0.65, *P* = 0.005), tumor circumferential extent (OR, 0.19, *P* = 0.036), distance from the anal verge (OR, 0.82, *P* = 0.019), and the neoadjuvant treatment regimen (OR, 9.33, *P* < 0.001) were significantly associated with pCR following neoadjuvant treatment. The tumor length (OR, 0.78, *P* = 0.015), tumor circumferential extent (OR = 0.15, *P* = 0.001), distance from the anal verge (OR, 0.56, *P* = 0.032), clinical T category (OR, 0.38, *P* = 0.012), and the neoadjuvant treatment regimen (OR, 2.90, *P* = 0.001) were significantly associated with good response (ypT0–2N0M0). CEA showed borderline significance for good response prediction (OR, 0.57, *P* = 0.055) in logistic-regression analysis (Table 3). Due to a confirmed predictive value of CEA in most of the previous rectal-cancer studies, CEA was manually selected as one of the predictive biomarkers in the prognostic model for good response.

**Table 2.** Univariate analysis of pretreatment parameters for pCR and tumor-downstaging prediction in 309 patients with locally advanced rectal cancer

Parameter	pCR (*n* = 53, %)	Non-pCR (*n* = 256, %)	*P*	Tumor downstaging (*n* = 132, %)	Poor response (*n* = 177, %)	*P*
Age, years			0.072			0.143
<55	32 (60.4)	119 (46.5)		71 (53.8)	80 (45.2)	
≥55	21 (39.6)	137 (53.5)		61 (46.2)	97 (54.8)	
Sex			0.633			0.182
Male	34 (64.2)	173 (67.6)		83 (62.9)	124 (70.1)	
Female	19 (35.8)	83 (32.4)		49 (37.1)	53 (29.9)	
Clinical T category (cT)			0.541			< 0.001
T2	2 (3.8)	4 (1.6)		5 (3.8)	1 (0.6)	
T3	43 (81.1)	208 (81.3)		116 (87.9)	135 (76.3)	
T4	8 (15.1)	44 (17.2)		11 (8.3)	41 (23.2)	
Clinical N category (cN)			0.340			0.192
N0	12 (22.6)	67 (26.2)		35 (26.5)	44 (24.9)	
N1	26 (49.1)	98 (38.3)		59 (44.7)	65 (36.7)	
N2	15 (28.3)	91 (35.5)		38 (28.8)	68 (38.4)	
TNM staging			0.592			0.743
Stage II	12 (22.6)	67 (26.2)		35 (26.5)	44 (24.9)	
Stage III	41 (77.4)	189 (73.8)		97 (73.5)	133 (75.1)	
CEA, ng/mL			0.797			0.001
≥5	13 (24.5)	85 (33.2)		29 (22.0)	69 (39.0)	
<5	40 (75.5)	171 (73.8)		103 (78.0)	108 (61.0)	
Tumor length, cm			< 0.001			< 0.001
≤4	39 (73.6)	117 (45.7)		81 (61.4)	75 (42.4)	
>4	14 (26.4)	139 (54.3)		51 (38.6)	102 (57.6)	
DTAV, cm			< 0.001			0.031
<4.5	31 (58.5)	77 (30.1)		55 (41.7)	53 (30.0)	
≥4.5	22 (41.5)	179 (69.9)		77 (58.3)	124 (70.0)	
TCE, circle			0.005			< 0.001
≤0.5	26 (49.1)	75 (29.3)		60 (45.5)	41 (23.2)	
>0.5	27 (50.9)	181 (70.7)		72 (54.5)	136 (76.8)	
MRF involvement			0.557			0.052
Positive	15 (28.3)	83 (32.4)		34(25.8)	64 (36.2)	
Negative	38 (71.7)	173 (67.6)		98 (74.2)	113 (63.8)	
Neoadjuvant regimen			< 0.001			0.007
5-Fluorouracil+RT	11 (20.8)	80 (31.3)		32 (24.2)	59 (33.3)	
mFOLFOX6+RT	34 (64.2)	65 (25.4)		55 (41.7)	44 (24.9)	
mFOLFOX6	8 (15.1)	111 (43.4)		45 (34.1)	74 (41.8)	

pCR, pathologic complete response; DTAV, distance from the anal verge; TCE, tumor circumferential extent; CEA, carcinoembryonic antigen; MRF, mesorectal fascia; RT, radiotherapy.

**Table 3.** Logistic-regression analysis for pCR and tumor-downstaging prediction

Prarameter	pCR		Tumor downstaging (ypT0–2N0)	
	OR (95% CI)	*P*	OR (95% CI)	*P*
Tumor length	0.65 (0.48–0.88)	0.005	0.78 (0.64–0.95)	0.015
TCE	0.19 (0.04–0.86)	0.036	0.15 (0.05–0.48)	0.001
DTAV	0.82 (0.69–0.96)	0.019	0.56 (0.33–0.95)	0.032
Clinical T category	–	–	0.38 (0.12–0.81)	0.012
Neoadjuvant regimen				
mFOLFOX6	1		1	
5-Fluorouracil+RT	2.22 (0.81–6.11)	0.120	1.17 (0.62–2.21)	0.635
mFOLFOX6+RT	9.33 (3.80–22.95)	<0.001	2.90 (1.57–5.38)	0.001
CEA	–	–	0.57 (0.32–1.01)	0.055

OR, odds ratios; CI, confidential interval; pCR, pathologic complete response; DTAV, distance from the anal verge; TCE, tumor circumferential extent; RT, radiotherapy; CEA, carcinoembryonic antigen.

### Construction of a nomogram for pCR and good response

**Figure 1.**
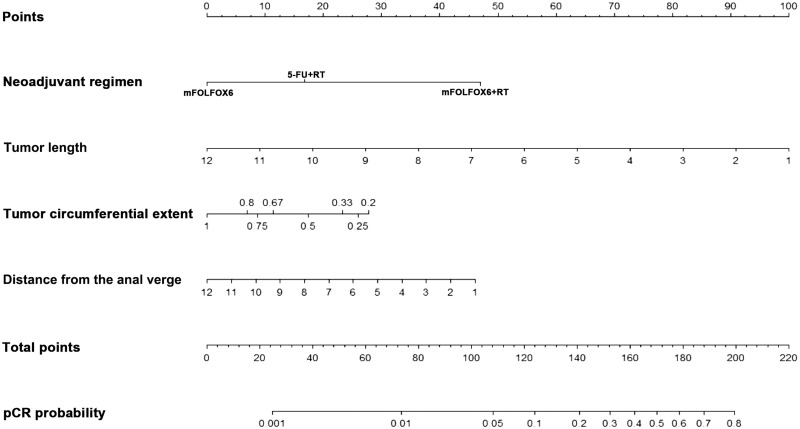
Nomogram for pathological complete response (pCR) prediction. A score for each predictor can be read out at the top scale (score). All summed scores can be converted directly to the probability of response.

**Figure 2.**
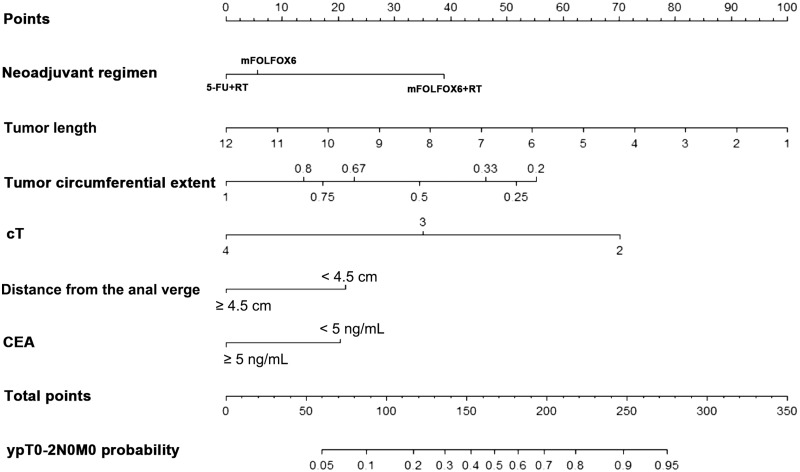
Nomogram for good response prediction. A score for each predictor can be read out at the top scale (score). All summed scores can be converted directly to the probability of response.

A nomogram incorporating significant predictors of logistic-regression analysis was developed to predict pCR and good response in LARC patients following neoadjuvant treatment (Figures 1 and 2). Each of these variables was assigned a score based on the point scale. After adding the total score, a vertical line was drawn downwards from the total point scale to obtain the probability of pCR and good response (see the bottom scale).

**Figure 3.**
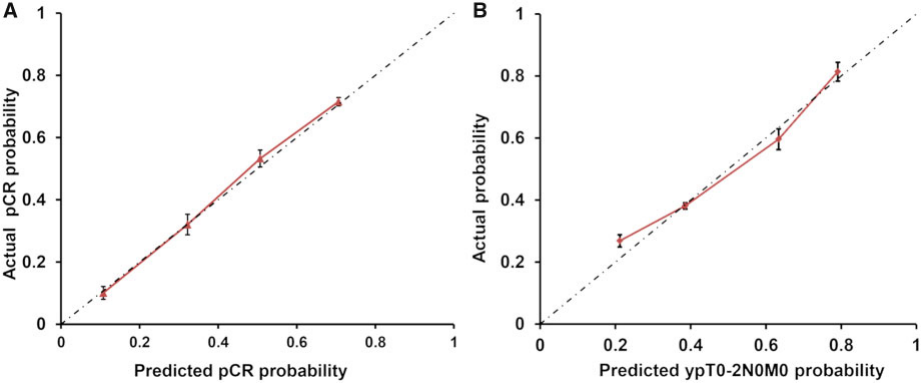
Calibration plots of the predicted and observed probabilities of pathological complete response (pCR) and good response (ypT0-2N0M0). (A) The prediction calculated using the nomograms is plotted on the *x*-axis and the observed rate of pCR is plotted on the *y*-axis. (B) The prediction calculated using the nomograms is plotted on the *x*-axis and the observed rate of tumor downstaging is plotted on the *y*-axis.

The nomogram was internally validated by using a bootstrap method with 1,000 resamples. The calibration plots showed good statistical performance upon internal validation between the nomogram prediction model and the actual observation for probability of pCR and good response (Figure 3). The C-index of the nomogram for predicting pCR was 0.802 (95% confidential interval [CI], 0.736–0.867) and that for good response was 0.730 (95% CI, 0.672–0.784).

## Discussion

Based on a phase III randomized clinical trial that compared fluorouracil-radiotherapy, mFOLFOX6 plus radiotherapy, and mFOLFOX6 chemotherapy alone as neoadjuvant treatment, a model for predicting pCR and good response (ypT0-2N0M0) has been developed in LARC patients under different neoadjuvant treatment regimens with pretreatment parameters. With the help of this model, response prediction was done before initiating the treatment, so that the patient can be offered the optimal treatment with a high success rate.

In this study, the efficacy was measured in two ways: pCR and good response (ypT0-2N0M0). Of the two endpoints, pCR is known to be the most robust factor for early efficacy and long-term survival in LARC patients. Pooled analysis of individual patient data from 17 different datasets showed a clear prognostic value of pCR after neoadjuvant treatment for long-term outcomes [10]. Also, tumor downstaging (ypT0-2N0M0) showed better survival in patients than in those without downstaging (ypStage II-III) [5]. This assists in predicting the group with good responders, as they could undergo a less invasive surgery, like transanal resection or transanal endoscopic microsurgical (TEM) excision.

In this predictive model, tumor length was regarded as the most important independent predictor for pCR and good response. Stiphout *et al.* [11] have developed another predictive model for pCR in rectal-cancer patients including the sequential PET-CT imaging and the results showed that tumor length was the most common factor associated with pCR. The present study also showed that smaller tumor size was predicted to be associated with an increased rate of pCR and tumor downstaging, which was in accordance with the results of the previous study. In addition, another two randomized clinical trials have reported that a 2- to 3-cm reduction in tumor size was usually required for patients to qualify for local excision after neoadjuvant chemoradiotherapy [12, 13].

Besides, the tumor circumferential extent and the distance of tumor from anal verge were also considered important predictors of pCR and good response. Yan *et al.* [14] have demonstrated that a tumor circumferential extent >50% was significantly associated with a poor pathologic tumor response. Das *et al.* [15] retrospectively analysed 562 rectal-cancer patients and found that greater tumor circumferential extent and greater distance of the tumor from the anal verge were independent predictors of low rates of pCR and downstaging. Overall, this meant that the dominant tumor dimension or tumor burden was the most predictive variable set for pCR and tumor downstaging.

CEA was also included in the model as one of the predictors of good response. This tumor biomarker has been widely used for predicting the response to neoadjuvant treatment in LARC patients. Although the significance was near the decision boundary (*P* = 0.055) in the present study, incorporation of CEA brings additional accuracy to the predictive model. The prognostic value of CEA in colorectal cancer was independent of clinical stage and differentiation grade [16]. Yoon *et al.* [17] have analysed a group of 351 rectal-cancer patients and demonstrated that the pretreatment CEA level is the most important clinical predictor of pathologic tumor response. In multivariate analysis, CEA levels of ≤5 μg/L were predictors of tumor downstaging. Park *et al.* [18] have conducted a retrospective analysis in 352 rectal-cancer patients and the results revealed that lower pretreatment CEA had a significant predictive value for good response. Buijsen *et al.* [19] have analysed the predictive value of blood biomarkers in a prospective study. The study analysed CEA as a continuous variable and demonstrated a significant predictive value for good tumor response.

Additionally, a neoadjuvant treatment regimen was also regarded as a predictive variable in this model, which was different from that of the previous predictive models for rectal-cancer patients and only fluoropyrimidine-based chemoradiotherapy was involved. Previously, several large prospective trials that added oxaliplatin as a radio-sensitizer showed increased acute toxicity but failed to increase the pCR rate or to improve the survival [20–22]. However, in the FOWARC study, a full dose of mFOLFOX6-chemoradiotherapy led to higher pCR rate and tumor-downstaging proportion, although the grade 3/4 toxicity was slightly higher than that of the other groups. The high pCR rate with a full dose of FOLFOX or CapeOX (Capecitabine and oxaliplatin) has been confirmed by several other phase II studies [23, 24]. With the incorporation of neoadjuvant regimens in the model, the probability of pCR and tumor downstaging with different regimens at the beginning of the therapy could be estimated and a better choice of treatment can be chosen. For those who could achieve a good response with neoadjuvant chemotherapy alone, radiotherapy might be avoided and the adverse events caused due to radiation exposure could be omitted [25].

The nomogram based on the clinical parameters has a reliable C-index on internal validation. The distribution of probability of a pCR or good response provided by the nomogram represented the true distribution in the data, which was confirmed by overall calibration.

The advantage of this study was that prediction can be done before undergoing any treatment and the regimen for neoadjuvant treatment could be chosen. More predictors should be added to increase the performance of the model in the future, including imaging variables such as the apparent diffusion coefficient or the T2 mapping of MRI [26–28] and biological variables such as gene signatures [29]. Another possibility to strengthen the predictive model is to incorporate response data that are obtained early during the neoadjuvant treatment or before surgery, which might help when making decisions of undergoing less invasive surgery or a ‘watch and wait’ strategy.

However, the main limitation of this study was that the nomogram was based on prospective clinical-trial data, with the inclusion of small sample sizes for each regimen. External validation of this prediction model is necessary for reproducibility of the model. We hope that our experience assists in accurately predicting pCR and tumor downstaging. Further study should focus on validating this model, both on external validation from other institutions and incorporation of other predictors in the model.

In conclusion, an accurate prediction model for pCR and good response in LARC patients based on a large prospective study was developed. To our knowledge, this is the first study to incorporate a neoadjuvant regimen in the model. This personalized treatment approach is expected to promote more complete responders and reduce the number of surgeries and any related complications, avoiding unnecessary toxicity. The model provides valuable decision support for more individualized treatment approaches in the future when validating prospectively.

## Authors' contributions

J.W.Z., Y.C., J.P.W., and Y.H.D. conceived of the study and participated in its design and coordination. X.Y.X., H.B.H., Z.H.W., and J.Y.L. performed the clinical data collection. P.L., X.J.W., M.J.H., H.W., and L.K. selected the patients. Z.Y.Z. re-evaluated the imaging of all the patients. J.W.Z. performed the statistical analyses and interpretation and drafted the manuscript. All authors read and approved the final manuscript.

## Funding

The study was supported by Science and Technology Program of Guangzhou [NO. 201803010073]. 

## Conflicts of interest

None declared.
